# Call to Eliminate Cervical Cancer in the Next Decade with a Focus on Brazil

**DOI:** 10.1055/s-0041-1722939

**Published:** 2021-01-29

**Authors:** Walquíria Quida Salles Pereira Primo, Neila Maria de Góis Speck, Cecilia Maria Roteli-Martins, César Eduardo Fernandes, Agnaldo Lopes da Silva Filho

**Affiliations:** 1 Universidade de Brasília, Brasília, DF, Brazil; 2 Escola Paulista de Medicina, Universidade Federal de São Paulo, São Paulo, SP, Brazil; 3 Faculdade de Medicina do ABC, Santo André, SP, Brazil; 4 Universidade Federal de Minas Gerais, Belo Horizonte, MG, Brazil

At the 73 ^rd^ General Assembly, in 2020, the World Health Organization (WHO) approved the adoption of the “Global Strategy to Accelerate the Elimination of Cervical Cancer as a Public Health Problem,” based on the guarantee of the three following pillars: 1) that 90% of girls up to 15 years of age receive the vaccine against human papillomavirus (HPV); 2) that 70% of women perform a screening with HPV testing at 35 and 45 years old; and 3) that 90% of women identified with precursor lesions or invasive cancer receive treatment. 1

Cervical cancer is a preventable, curable disease with high morbidity and mortality among women in countries without organized prevention programs, like Brazil. Globally, more than 570,000 new cases emerge annually, and more than 311,000 women die each year. According to the WHO, most of these deaths occur in countries with low development rates. In Brazil, cervical cancer ranks 3rd among malignant neoplasms in women, with 15.43 cases per 100,000 women per year, and 4th in mortality. 1 2

In global actions of screening for this type of cancer in women aged 25 to 64 years, testing is performed in an organized way, with cervicovaginal oncotic cytology and triennial interval covering 70% of this population, thus resulting in a significant reduction in mortality, with figures under 2 deaths per 100,000 women a year. However, the sensitivity of cytology has been questioned, and the screening with HPV testing has shown greater accuracy. In the WHO's commitment to eliminate cervical cancer, the proposal of screening with HPV testing of 70% of women at 2 points in time, at the ages of 35 and 45 years, will reduce the mortality by about a third.

In Brazil, screening is not organized in the public system and is done exclusively by oncotic cytology in an opportunistic way. The screening guidelines published in 2016 by the Ministry of Health recommend the oncotic cytology examination at a 3-year interval for women aged 25 to 64 years old who had 2 previous normal results. Considering a target population of 50 million women and a 3-year interval, the screening would reach 17 million women a year. Data from the National Health Service (SUS, in the Portuguese acronym) show that 10 million women outside of the target population are screened a year. According to the publication by Costa et al. (2015) 3 on data regarding oncotic cytology exams collected by the Ministry of Health in the years 2012 to 2013:

- Coverage is below 70%;
- 50% of women are screened annually;
- 10% of women are screened every 3 years;
- 20% of screenings are performed in women under 25 years and even under 20 years of age.

The HPV vaccines are highly effective, promote a significant decrease in HPV infections and, consequently, in neoplastic cervical lesions. However, in Brazil, vaccination coverage has been below what is necessary for effective action in the coming decades. The reasons explaining the low coverage are mainly the logistical barriers of access and lack of continuous education of the population.

In 2014, the National Immunization Program (NIP) introduced the quadrivalent vaccine for girls aged 9 to 14 years old on a 2-dose schedule at a 6-month interval. In 2017, the program also included 11 to 14 years old boys on a 2-dose schedule. For the first dose, in the first year of implementation, coverage was above 80%. For the second dose, when the government transferred vaccination from the school environment to health facilities, there was a significant drop in coverage. In subsequent years, coverage has continued dropping, reflecting the little interest during this important time of pandemic. The insufficient coverage for boys demonstrates the need for strategies by professionals, educators, and parents to expand the reach of vaccination programs.

In the state of Amazonas, where the incidence of cervical cancer is 50/100,000 women, coverage is currently insufficient for both girls and boys at all ages. In 2014, the goal of covering the first dose of HPV vaccines was achieved in just 3 months, but experience has shown that vaccinating adolescents against HPV outside the school environment makes the goal of eradicating cervical cancer in the next decade more difficult.

In 2020, from January to July, mortality from invasive cervical cancer was between 25 and 30 deaths per month (1 death every 24 hours) across the state of Amazonas, and vaccine coverage fell to concerning levels. The government of Amazonas suspended treatment procedures due to the pandemic, and a major impact on mortality is expected in the coming months.

Cervical cancer is an important public health problem in Brazil and worldwide, even though it is a preventable neoplasm with the adoption of primary prevention (HPV vaccine) and secondary prevention (screening tests) measures that have proven efficacy and effectiveness. Cervical cancer is a preventable disease that if diagnosed early, has a high cure rate. Adequate treatment without delays is an important prognostic factor that has already been proven in numerous studies.

Invasive cervical cancers are usually treated with surgery or radiation combined with chemotherapy. The choice of the best therapeutic option depends on the clinical stage of the tumor, age, reproductive history, general condition of the patient, and conditions available at the local health service. In Brazil, ∼ 80% of women diagnosed with invasive disease are in an advanced stage and are, thus, ineligible for surgery, thereby requiring radiotherapy and chemotherapy as treatment and generating an enormous social and financial cost. 4

The consequence of socioeconomic and cultural characteristics found in Brazil is a scenario in which factors related to poverty and development coexist. Thus, the access to screening and diagnosis services and timely treatments is severely compromised. Consequently, limitations of access to health services not only prevent poor women from being diagnosed, but also deprive them from having adequate and sufficient treatment in time to obtain a cure. In Brazil, this type of cancer occurs predominantly in non-white women (62.7%) with low educational level (62.1%), reinforcing that this neoplasia is not only a health issue but also a social problem. 5

The Brazilian public health system serves 75.32% of cancer patients, that is, ∼ 448,959 cancer cases were treated in the public health system in 2016. The period between diagnosis and the 1 ^st^ treatment was longer than 60 days in 58.0% of cases, and early deaths occurred in 11% of them. 5 Note that radiotherapy plays an important role as an adjuvant or exclusive treatment, especially in cervical cancer, depending on the extent of the disease, and in Brazil there is only 50.8% of the necessary amount of radiotherapy treatment equipment. 6

Regarding deaths, almost 9 out of 10 deaths from cervical cancer occur in less developed regions, where the risk of dying from cervical cancer before the age of 75 is 3 times higher. We highlight the preventable deaths of young women—at the peak of their working lives and many of whom are head of their household—if they are treated properly.

The purpose of this call is to focus attention on the serious health problem of cervical cancer, which, being asymptomatic in the early stages, is unable to mobilize the public opinion effectively in order that the tools available for its eradication are applied.
